# Crystal structure of ethyl 4-(2,4-di­chloro­phen­yl)-2-methyl-4*H*-benzo[4,5]thia­zolo[3,2-*a*]pyrimidine-3-carboxyl­ate

**DOI:** 10.1107/S2056989015007033

**Published:** 2015-04-15

**Authors:** T. Sankar, S. Naveen, N. K. Lokanath, K. Gunasekaran

**Affiliations:** a Centre of Advanced Study in Crystallography and Biophysics, University of Madras, Guindy Campus, Chennai 600 025, India; b Institution of Excellence, University of Mysore, Manasagangotri, Mysore 570 006, India; c Department of Studies in Physics, University of Mysore, Manasagangotri, Mysore 570 006, India

**Keywords:** crystal structure, pyrimidine, benzo­thia­zole, C—H⋯O hydrogen bonds, C—H⋯N hydrogen bonds

## Abstract

In the title compound, C_20_H_16_Cl_2_N_2_O_2_S, the pyrimidine ring has a screw-boat conformation. The attached di­chloro­phenyl ring is twisted at an angle of 89.29 (13)° with respect to the pyrimidine ring mean plane. The benzo­thia­zole group is approximately planar (r.m.s. deviation = 0.008 Å) and inclined to the pyrimidine ring mean plane by 3.04 (10)°. The carboxyl­ate group assumes an extended conformation with respect to the pyrimidine ring, which can be seen from the O=C—O—C torsion angle of 3.2 (4) °. In the crystal, mol­ecules are linked *via* C—H⋯O and C—H⋯N hydrogen bonds, forming slabs lying parallel to (100).

## Related literature   

For general background and literature on the biological properties of pyrimidine derivatives, see: Kumar *et al.* (2002 ▸; Baraldi *et al.* (2002 ▸); Nasr & Gineinah (2002 ▸).
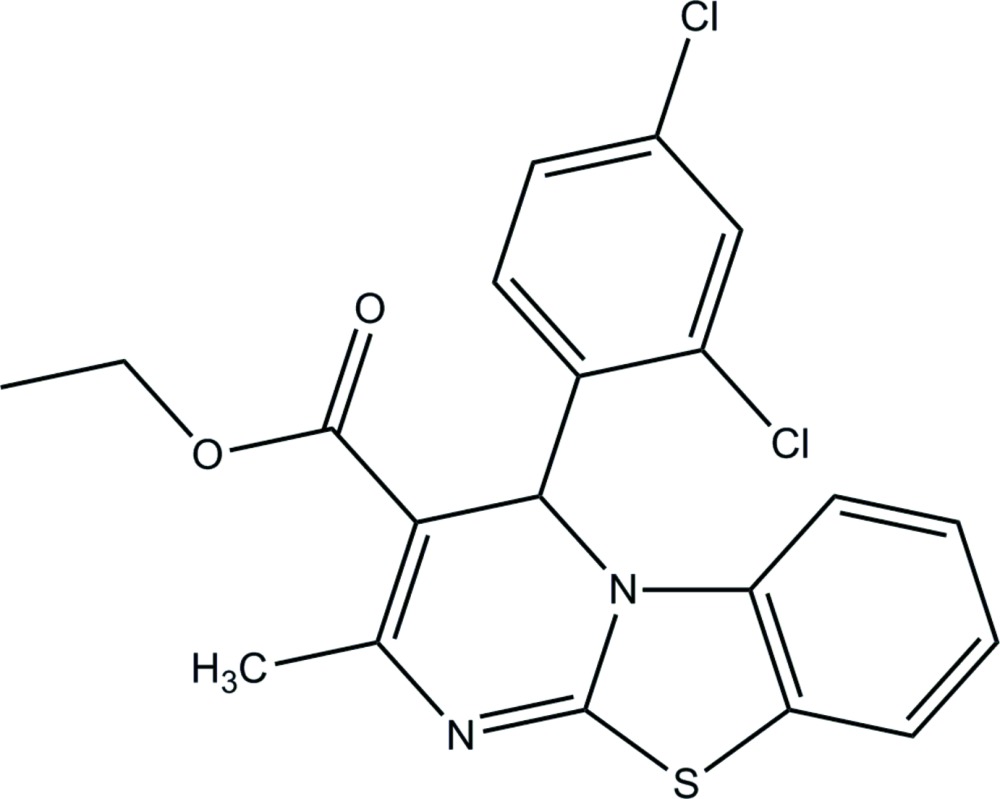


## Experimental   

### Crystal data  

C_20_H_16_Cl_2_N_2_O_2_S
*M* *_r_* = 419.31
Monoclinic, 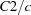

*a* = 38.654 (8) Å
*b* = 11.787 (3) Å
*c* = 8.774 (2) Å
β = 102.415 (14)°
*V* = 3904.1 (15) Å^3^
*Z* = 8
Cu *K*α radiation
μ = 4.14 mm^−1^
*T* = 296 K
0.30 × 0.27 × 0.25 mm

### Data collection  

Bruker SMART APEXII CCD diffractometer
Absorption correction: multi-scan (*SADABS*; Bruker, 2008 ▸) *T*_min_ = 0.370, *T*_max_ = 0.424
10089 measured reflections
3114 independent reflections
2519 reflections with *I* > 2σ(*I*)
*R*_int_ = 0.042

### Refinement  

*R*[*F* ^2^ > 2σ(*F* ^2^)] = 0.047
*wR*(*F* ^2^) = 0.139
*S* = 1.04
3114 reflections
246 parameters
H-atom parameters constrained
Δρ_max_ = 0.36 e Å^−3^
Δρ_min_ = −0.31 e Å^−3^

### 

Data collection: *APEX2* (Bruker, 2008 ▸); cell refinement: *SAINT* (Bruker, 2008 ▸); data reduction: *SAINT*; program(s) used to solve structure: *SHELXS97* (Sheldrick, 2008 ▸); program(s) used to refine structure: *SHELXL97* (Sheldrick, 2008 ▸); molecular graphics: *ORTEP-3 for Windows* (Farrugia, 2012 ▸) and *Mercury* (Macrae *et al.*, 2008 ▸); software used to prepare material for publication: *SHELXL97* and *PLATON* (Spek, 2009 ▸).

## Supplementary Material

Crystal structure: contains datablock(s) global, I. DOI: 10.1107/S2056989015007033/su5114sup1.cif

Structure factors: contains datablock(s) I. DOI: 10.1107/S2056989015007033/su5114Isup2.hkl

Supporting information file. DOI: 10.1107/S2056989015007033/su5114Isup3.cml

**Fig. 1.**
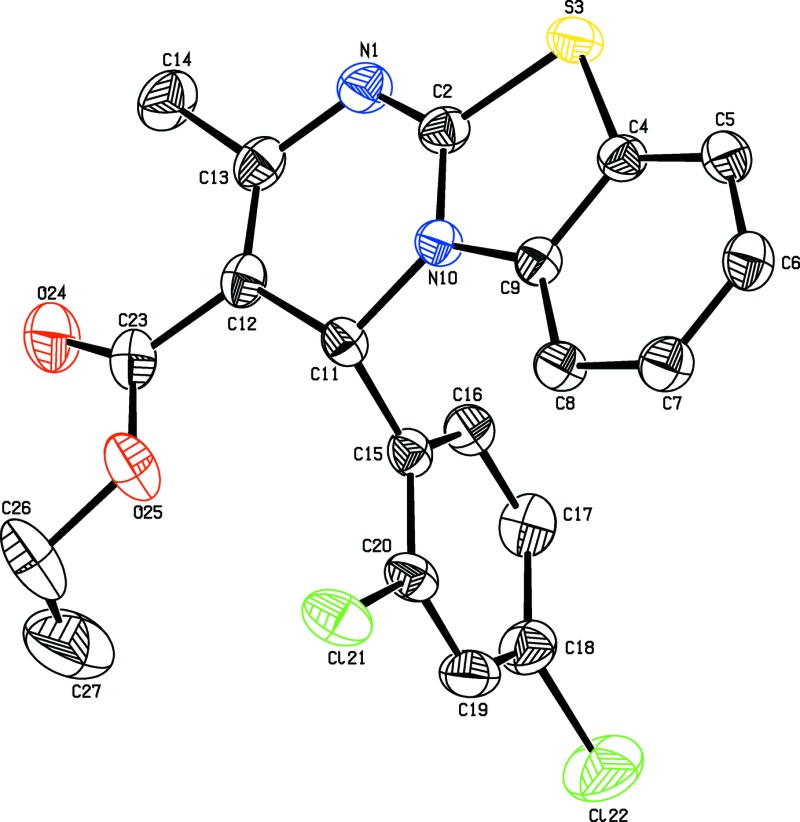
The molecular structure of the title compound, with atom labelling. Displacement ellipsoids are drawn at the 30% probability level.

**Fig. 2.**
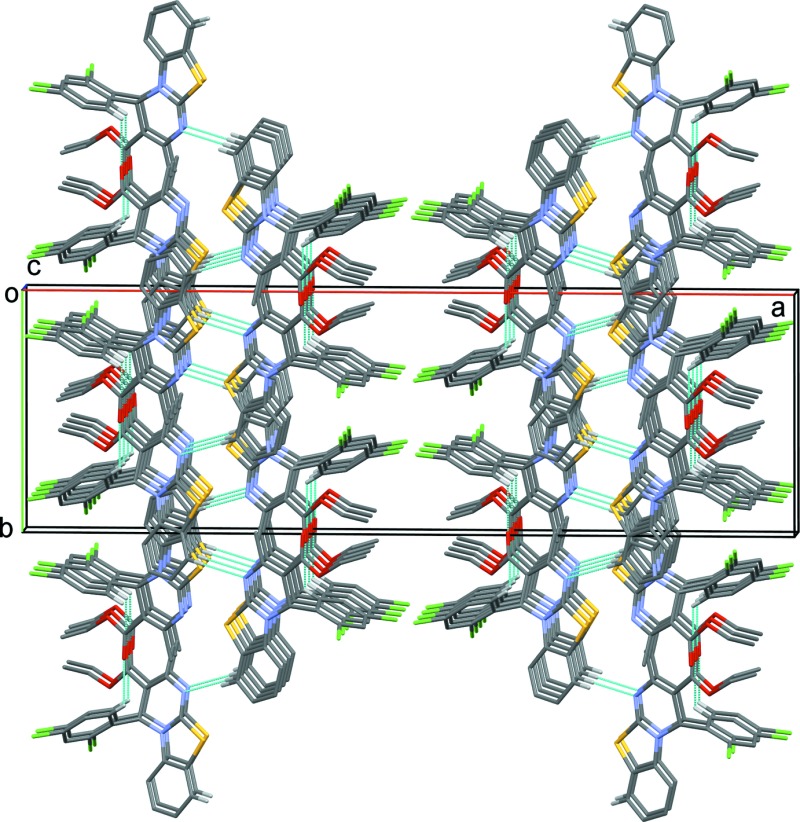
The crystal packing of the molecules viewed down the c axis.

. DOI: 10.1107/S2056989015007033/su5114fig1.tif
The mol­ecular structure of the title compound, with atom labelling. Displacement ellipsoids are drawn at the 30% probability level.

c . DOI: 10.1107/S2056989015007033/su5114fig2.tif
The crystal packing of the mol­ecules viewed down the *c* axis.

CCDC reference: 1056200

Additional supporting information:  crystallographic information; 3D view; checkCIF report

## Figures and Tables

**Table 1:** Hydrogen-bond geometry (, )

*D*H*A*	*D*H	H*A*	*D* *A*	*D*H*A*
C5H5N1^i^	0.93	2.59	3.515(4)	176
C16H16O24^ii^	0.93	2.53	3.180(4)	128

Symmetry codes: (i) 
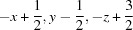
; (ii) 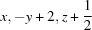.

## supplementary crystallographic information

### S1. Structural commentary

Many pyrimidine derivatives are reported to have anti-bacterial, anti-tumor and anti-viral activities (Kumar *et al.*, 2012; Baraldi *et al.*, 2002; Nasr *et al.*, 2002). In view of their important properties we synthesized the title compound and report herein on its crystal structure.

The molecular structure of the title compound is shown in Fig. 1. The pyrimidine ring [N1/C13/C12/C11/N10/C2] has a screw-boat conformation and its mean plane is inclined to the planar benzo­thia­zole group [S3/C2/N10/C4—C9; r.m.s. deviation = 0.008 Å] by 3.04 (10) °. The di­chloro­phenyl ring [C15—C20] is twisted at an angle of 89.29 (13) ° with respect to the pyrimidine ring mean plane. The carboxyl­ate group assumes an extended conformation with respect to the pyrimidine ring, which can be seen from the torsion angle O24—C23—O25—C26 = 3.2 (4) °.

In the crystal, molecules are linked via C—H···O and C—H···N hydrogen bonds forming slabs lying parallel to (100); see Table 1 and Fig. 2.

### S2. Synthesis and crystallization

A mixture of 2,4-di­chloro benzaldehyde was treated with ethyl aceto­acetate and 2-amino­benzo­thia­zole in the presence of ammonium acetate in ethanol. The mixture was gently warmed in a water bath at 353 K until the colour changed and it was then kept aside overnight at room temperature. The completion of reaction was monitored by TLC. The solid obtained was separated and purified by means of column chromatography using hexane and ethyl acetate as eluent. The sample was recrystallized using a 1:1 mixture of ethanol and THF, yielding yellow block-like crystals.

### S3. Refinement

Crystal data, data collection and structure refinement details are summarized in Table 2. H atoms were positioned geometrically and allowed to ride on their parent atoms: C—H = 0.93-0.98 Å with U_iso_(H) = 1.5U_eq_(C) for methyl H atoms and 1.2U_eq_(C) for other H atoms.

### Crystal data

**Table:**

C_20_H_16_Cl_2_N_2_O_2_S	*F*(000) = 1728
*M**_r_* = 419.31	*D*_x_ = 1.427 Mg m^−^^3^
Monoclinic, *C*2/*c*	Cu *K*α radiation, λ = 1.54178 Å
Hall symbol: -C 2yc	Cell parameters from 3114 reflections
*a* = 38.654 (8) Å	θ = 5.1–64.1°
*b* = 11.787 (3) Å	µ = 4.14 mm^−^^1^
*c* = 8.774 (2) Å	*T* = 296 K
β = 102.415 (14)°	Block, yellow
*V* = 3904.1 (15) Å^3^	0.30 × 0.27 × 0.25 mm
*Z* = 8	

### Data collection

**Table:**

Bruker SMART APEXII CCD diffractometer	3114 independent reflections
Radiation source: fine-focus sealed tube	2519 reflections with *I* > 2σ(*I*)
Graphite monochromator	*R*_int_ = 0.042
ω and φ scans	θ_max_ = 64.1°, θ_min_ = 5.1°
Absorption correction: multi-scan (*SADABS*; Bruker, 2008)	*h* = −43→44
*T*_min_ = 0.370, *T*_max_ = 0.424	*k* = −12→12
10089 measured reflections	*l* = −10→9

### Refinement

**Table:**

Refinement on *F*^2^	Primary atom site location: structure-invariant direct methods
Least-squares matrix: full	Secondary atom site location: difference Fourier map
*R*[*F*^2^ > 2σ(*F*^2^)] = 0.047	Hydrogen site location: inferred from neighbouring sites
*wR*(*F*^2^) = 0.139	H-atom parameters constrained
*S* = 1.04	*w* = 1/[σ^2^(*F*_o_^2^) + (0.0772*P*)^2^ + 2.6744*P*] where *P* = (*F*_o_^2^ + 2*F*_c_^2^)/3
3114 reflections	(Δ/σ)_max_ < 0.001
246 parameters	Δρ_max_ = 0.36 e Å^−^^3^
0 restraints	Δρ_min_ = −0.31 e Å^−^^3^

### Special details

**Table:**

Geometry. All e.s.d.'s (except the e.s.d. in the dihedral angle between two l.s. planes) are estimated using the full covariance matrix. The cell e.s.d.'s are taken into account individually in the estimation of e.s.d.'s in distances, angles and torsion angles; correlations between e.s.d.'s in cell parameters are only used when they are defined by crystal symmetry. An approximate (isotropic) treatment of cell e.s.d.'s is used for estimating e.s.d.'s involving l.s. planes.
Refinement. Refinement of *F*^2^ against ALL reflections. The weighted *R*-factor *wR* and goodness of fit *S* are based on *F*^2^, conventional *R*-factors *R* are based on *F*, with *F* set to zero for negative *F*^2^. The threshold expression of *F*^2^ > σ(*F*^2^) is used only for calculating *R*-factors(gt) *etc*. and is not relevant to the choice of reflections for refinement. *R*-factors based on *F*^2^ are statistically about twice as large as those based on *F*, and *R*- factors based on ALL data will be even larger.

### Fractional atomic coordinates and isotropic or equivalent isotropic displacement parameters (Å^2^)

**Table:**

	*x*	*y*	*z*	*U*_iso_*/*U*_eq_	
N1	0.29291 (6)	0.8365 (2)	0.6499 (3)	0.0540 (6)	
C2	0.29669 (6)	0.7278 (2)	0.6518 (3)	0.0450 (6)	
S3	0.267722 (17)	0.63964 (6)	0.72345 (9)	0.0552 (3)	
C4	0.28999 (6)	0.5193 (2)	0.6806 (3)	0.0446 (6)	
C5	0.28233 (7)	0.4079 (2)	0.7057 (3)	0.0520 (7)	
H5	0.2631	0.3891	0.7488	0.062*	
C6	0.30359 (8)	0.3250 (3)	0.6658 (4)	0.0561 (7)	
H6	0.2990	0.2492	0.6832	0.067*	
C7	0.33195 (8)	0.3533 (2)	0.5997 (4)	0.0560 (7)	
H7	0.3460	0.2958	0.5730	0.067*	
C8	0.33984 (7)	0.4649 (2)	0.5723 (3)	0.0484 (7)	
H8	0.3589	0.4831	0.5276	0.058*	
C9	0.31847 (6)	0.5488 (2)	0.6136 (3)	0.0406 (6)	
N10	0.32157 (5)	0.66662 (18)	0.5982 (2)	0.0417 (5)	
C11	0.35145 (6)	0.7226 (2)	0.5479 (3)	0.0411 (6)	
H11	0.3537	0.6908	0.4474	0.049*	
C12	0.34297 (7)	0.8489 (2)	0.5275 (3)	0.0463 (6)	
C13	0.31608 (7)	0.8979 (2)	0.5811 (3)	0.0513 (7)	
C14	0.30725 (9)	1.0217 (3)	0.5713 (4)	0.0694 (9)	
H14A	0.3263	1.0638	0.6345	0.104*	
H14B	0.2859	1.0344	0.6084	0.104*	
H14C	0.3038	1.0464	0.4649	0.104*	
C15	0.38581 (6)	0.7024 (2)	0.6676 (3)	0.0409 (6)	
C16	0.38873 (7)	0.7454 (2)	0.8175 (3)	0.0472 (6)	
H16	0.3695	0.7836	0.8417	0.057*	
C17	0.41907 (8)	0.7332 (3)	0.9307 (4)	0.0608 (8)	
H17	0.4205	0.7630	1.0300	0.073*	
C18	0.44751 (8)	0.6758 (3)	0.8946 (4)	0.0627 (8)	
C19	0.44582 (7)	0.6332 (3)	0.7486 (4)	0.0612 (8)	
H19	0.4651	0.5953	0.7251	0.073*	
C20	0.41531 (7)	0.6470 (2)	0.6369 (3)	0.0491 (7)	
Cl21	0.41503 (2)	0.59197 (7)	0.45153 (10)	0.0706 (3)	
Cl22	0.48585 (3)	0.65809 (11)	1.03772 (14)	0.1075 (4)	
C23	0.36748 (8)	0.9170 (3)	0.4592 (3)	0.0556 (7)	
O24	0.36870 (7)	1.0189 (2)	0.4524 (3)	0.0880 (8)	
O25	0.39033 (6)	0.8507 (2)	0.4043 (3)	0.0734 (7)	
C26	0.41805 (13)	0.9061 (4)	0.3433 (5)	0.1021 (15)	
H26A	0.4088	0.9754	0.2903	0.122*	
H26B	0.4253	0.8568	0.2674	0.122*	
C27	0.44862 (13)	0.9328 (5)	0.4666 (8)	0.134 (2)	
H27A	0.4416	0.9836	0.5400	0.201*	
H27B	0.4665	0.9682	0.4221	0.201*	
H27C	0.4579	0.8642	0.5190	0.201*	

### Atomic displacement parameters (Å^2^)

**Table:**

	*U*^11^	*U*^22^	*U*^33^	*U*^12^	*U*^13^	*U*^23^
N1	0.0535 (13)	0.0408 (15)	0.0701 (16)	0.0048 (10)	0.0185 (12)	−0.0012 (11)
C2	0.0429 (13)	0.0401 (17)	0.0513 (15)	0.0021 (11)	0.0086 (11)	−0.0025 (12)
S3	0.0456 (4)	0.0476 (5)	0.0771 (5)	0.0017 (3)	0.0239 (3)	0.0002 (3)
C4	0.0394 (12)	0.0424 (17)	0.0512 (15)	−0.0019 (11)	0.0077 (11)	0.0004 (12)
C5	0.0495 (15)	0.0455 (18)	0.0619 (17)	−0.0077 (13)	0.0142 (13)	0.0038 (13)
C6	0.0656 (18)	0.0351 (16)	0.0682 (19)	−0.0063 (13)	0.0155 (15)	0.0017 (13)
C7	0.0608 (17)	0.0400 (18)	0.0682 (19)	0.0019 (13)	0.0163 (15)	−0.0035 (14)
C8	0.0491 (14)	0.0408 (17)	0.0574 (17)	−0.0014 (12)	0.0162 (12)	−0.0049 (13)
C9	0.0420 (13)	0.0351 (15)	0.0429 (14)	−0.0045 (11)	0.0050 (11)	−0.0022 (11)
N10	0.0421 (11)	0.0343 (13)	0.0502 (12)	−0.0013 (9)	0.0136 (10)	−0.0016 (9)
C11	0.0462 (13)	0.0366 (15)	0.0432 (14)	−0.0043 (11)	0.0154 (11)	−0.0037 (11)
C12	0.0538 (15)	0.0379 (16)	0.0450 (15)	−0.0023 (11)	0.0058 (12)	0.0034 (11)
C13	0.0560 (16)	0.0381 (16)	0.0563 (17)	0.0024 (12)	0.0044 (13)	0.0004 (12)
C14	0.075 (2)	0.0404 (19)	0.092 (2)	0.0083 (15)	0.0153 (18)	0.0044 (16)
C15	0.0456 (13)	0.0315 (14)	0.0479 (14)	−0.0041 (11)	0.0152 (11)	−0.0003 (11)
C16	0.0526 (14)	0.0401 (16)	0.0513 (15)	0.0009 (12)	0.0165 (12)	−0.0034 (12)
C17	0.0724 (19)	0.057 (2)	0.0495 (17)	−0.0046 (16)	0.0048 (15)	−0.0073 (14)
C18	0.0493 (16)	0.063 (2)	0.070 (2)	−0.0035 (15)	0.0002 (14)	0.0069 (16)
C19	0.0433 (15)	0.057 (2)	0.085 (2)	0.0012 (13)	0.0171 (15)	0.0003 (17)
C20	0.0490 (14)	0.0414 (16)	0.0623 (17)	−0.0040 (12)	0.0241 (13)	−0.0068 (13)
Cl21	0.0700 (5)	0.0747 (6)	0.0771 (5)	−0.0009 (4)	0.0375 (4)	−0.0252 (4)
Cl22	0.0677 (6)	0.1307 (10)	0.1058 (8)	0.0037 (6)	−0.0221 (5)	0.0064 (7)
C23	0.0678 (18)	0.048 (2)	0.0501 (17)	−0.0049 (14)	0.0103 (14)	0.0078 (13)
O24	0.0960 (18)	0.0479 (16)	0.127 (2)	−0.0077 (12)	0.0385 (16)	0.0245 (14)
O25	0.0905 (16)	0.0654 (16)	0.0767 (15)	−0.0168 (12)	0.0455 (13)	−0.0005 (11)
C26	0.126 (3)	0.104 (3)	0.098 (3)	−0.039 (3)	0.071 (3)	−0.006 (2)
C27	0.088 (3)	0.143 (5)	0.182 (6)	−0.025 (3)	0.050 (4)	−0.008 (4)

### Geometric parameters (Å, º)

**Table:**

N1—C2	1.290 (4)	C14—H14A	0.9600
N1—C13	1.387 (4)	C14—H14B	0.9600
C2—N10	1.364 (3)	C14—H14C	0.9600
C2—S3	1.741 (3)	C15—C20	1.390 (3)
S3—C4	1.741 (3)	C15—C16	1.392 (4)
C4—C5	1.375 (4)	C16—C17	1.372 (4)
C4—C9	1.399 (3)	C16—H16	0.9300
C5—C6	1.369 (4)	C17—C18	1.384 (4)
C5—H5	0.9300	C17—H17	0.9300
C6—C7	1.387 (4)	C18—C19	1.365 (5)
C6—H6	0.9300	C18—Cl22	1.737 (3)
C7—C8	1.383 (4)	C19—C20	1.371 (4)
C7—H7	0.9300	C19—H19	0.9300
C8—C9	1.385 (4)	C20—Cl21	1.748 (3)
C8—H8	0.9300	C23—O24	1.204 (4)
C9—N10	1.403 (3)	C23—O25	1.343 (4)
N10—C11	1.478 (3)	O25—C26	1.452 (4)
C11—C15	1.524 (3)	C26—C27	1.455 (7)
C11—C12	1.527 (4)	C26—H26A	0.9700
C11—H11	0.9800	C26—H26B	0.9700
C12—C13	1.358 (4)	C27—H27A	0.9600
C12—C23	1.465 (4)	C27—H27B	0.9600
C13—C14	1.497 (4)	C27—H27C	0.9600
			
C2—N1—C13	116.2 (2)	C13—C14—H14B	109.5
N1—C2—N10	127.4 (2)	H14A—C14—H14B	109.5
N1—C2—S3	121.2 (2)	C13—C14—H14C	109.5
N10—C2—S3	111.4 (2)	H14A—C14—H14C	109.5
C4—S3—C2	91.26 (12)	H14B—C14—H14C	109.5
C5—C4—C9	121.3 (2)	C20—C15—C16	116.8 (2)
C5—C4—S3	127.6 (2)	C20—C15—C11	124.7 (2)
C9—C4—S3	111.0 (2)	C16—C15—C11	118.4 (2)
C6—C5—C4	118.6 (3)	C17—C16—C15	121.9 (3)
C6—C5—H5	120.7	C17—C16—H16	119.0
C4—C5—H5	120.7	C15—C16—H16	119.0
C5—C6—C7	120.5 (3)	C16—C17—C18	118.9 (3)
C5—C6—H6	119.8	C16—C17—H17	120.6
C7—C6—H6	119.7	C18—C17—H17	120.6
C8—C7—C6	121.6 (3)	C19—C18—C17	121.0 (3)
C8—C7—H7	119.2	C19—C18—Cl22	119.7 (3)
C6—C7—H7	119.2	C17—C18—Cl22	119.3 (3)
C7—C8—C9	117.9 (3)	C18—C19—C20	119.2 (3)
C7—C8—H8	121.1	C18—C19—H19	120.4
C9—C8—H8	121.1	C20—C19—H19	120.4
C8—C9—C4	120.0 (2)	C19—C20—C15	122.2 (3)
C8—C9—N10	127.8 (2)	C19—C20—Cl21	117.0 (2)
C4—C9—N10	112.2 (2)	C15—C20—Cl21	120.9 (2)
C2—N10—C9	114.1 (2)	O24—C23—O25	121.8 (3)
C2—N10—C11	121.4 (2)	O24—C23—C12	127.0 (3)
C9—N10—C11	124.0 (2)	O25—C23—C12	111.2 (3)
N10—C11—C15	110.2 (2)	C23—O25—C26	117.7 (3)
N10—C11—C12	108.0 (2)	O25—C26—C27	111.6 (4)
C15—C11—C12	111.5 (2)	O25—C26—H26A	109.3
N10—C11—H11	109.0	C27—C26—H26A	109.3
C15—C11—H11	109.0	O25—C26—H26B	109.3
C12—C11—H11	109.0	C27—C26—H26B	109.3
C13—C12—C23	121.3 (3)	H26A—C26—H26B	108.0
C13—C12—C11	122.4 (2)	C26—C27—H27A	109.5
C23—C12—C11	116.1 (2)	C26—C27—H27B	109.5
C12—C13—N1	122.9 (3)	H27A—C27—H27B	109.5
C12—C13—C14	125.2 (3)	C26—C27—H27C	109.5
N1—C13—C14	111.9 (3)	H27A—C27—H27C	109.5
C13—C14—H14A	109.5	H27B—C27—H27C	109.5

### Hydrogen-bond geometry (Å, º)

**Table:**

*D*—H···*A*	*D*—H	H···*A*	*D*···*A*	*D*—H···*A*
C5—H5···N1^i^	0.93	2.59	3.515 (4)	176
C16—H16···O24^ii^	0.93	2.53	3.180 (4)	128

Symmetry codes: (i) −*x*+1/2, *y*−1/2, −*z*+3/2; (ii) *x*, −*y*+2, *z*+1/2.

## References

[bb1] Baraldi, P. G., Pavani, M. G., Nuñez, M. del C., Brigidi, P., Vitali, B., Gambari, R. & Romagnoli, R. (2002). *Bioorg. Med. Chem.* **10**, 449–456. 10.1016/s0968-0896(01)00294-2 11741793

[bb2] Bruker (2008). *APEX2*, *SAINT* and *SADABS*. Bruker AXS Inc., Madison, Wisconsin, USA.

[bb3] Farrugia, L. J. (2012). *J. Appl. Cryst.* **45**, 849–854.

[bb4] Kumar, A., Sinha, S. & Chauhan, P. M. (2002). *Bioorg. Med. Chem. Lett.* **12**, 667-669. 10.1016/s0960-894x(01)00829-0 11844696

[bb5] Macrae, C. F., Bruno, I. J., Chisholm, J. A., Edgington, P. R., McCabe, P., Pidcock, E., Rodriguez-Monge, L., Taylor, R., van de Streek, J. & Wood, P. A. (2008). *J. Appl. Cryst.* **41**, 466–470.

[bb6] Nasr, M. N. & Gineinah, M. M. (2002). *Arch. Pharm. Pharm. Med. Chem.* **335**, 289–295. 10.1002/1521-4184(200208)335:6<289::AID-ARDP289>3.0.CO;2-Z 12210772

[bb7] Sheldrick, G. M. (2008). *Acta Cryst.* A**64**, 112–122. 10.1107/S0108767307043930 18156677

[bb8] Spek, A. L. (2009). *Acta Cryst.* D**65**, 148–155. 10.1107/S090744490804362X PMC2631630 19171970

