# Supplementary figures and images for: Crystal structure of ethyl 4-(2,4-di­chloro­phen­yl)-2-methyl-4H-benzo[4,5]thia­zolo[3,2-a]pyrimidine-3-carboxyl­ate
Source: Acta Crystallogr E Crystallogr Commun. 2015 Apr 15;71(Pt 5):o306–7. doi: 10.1107/S2056989015007033 (PMC4420040; doi:10.1107/S2056989015007033)

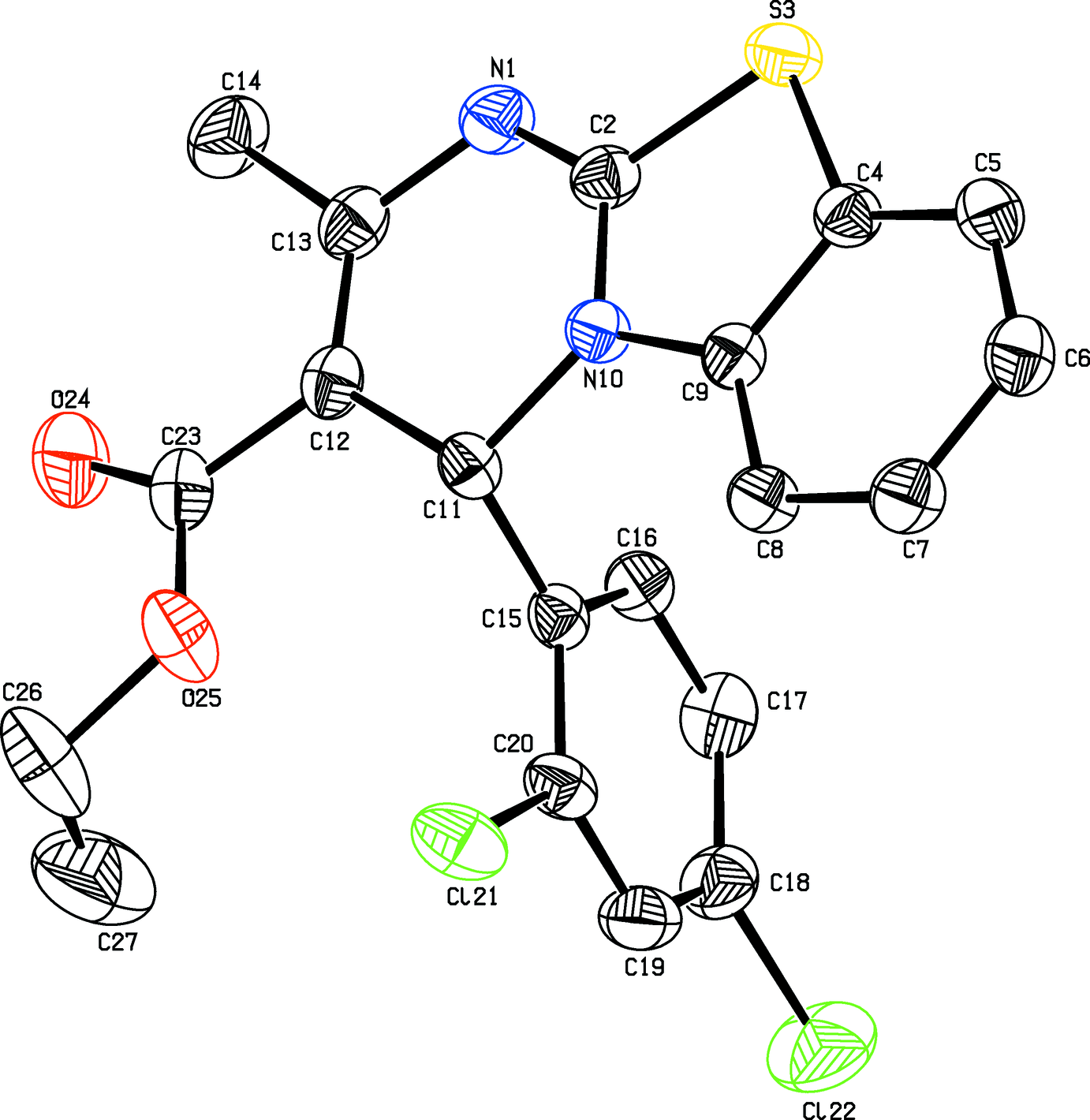

Supplement: Supplementary file 4 [file e-71-0o306-fig1.tif]

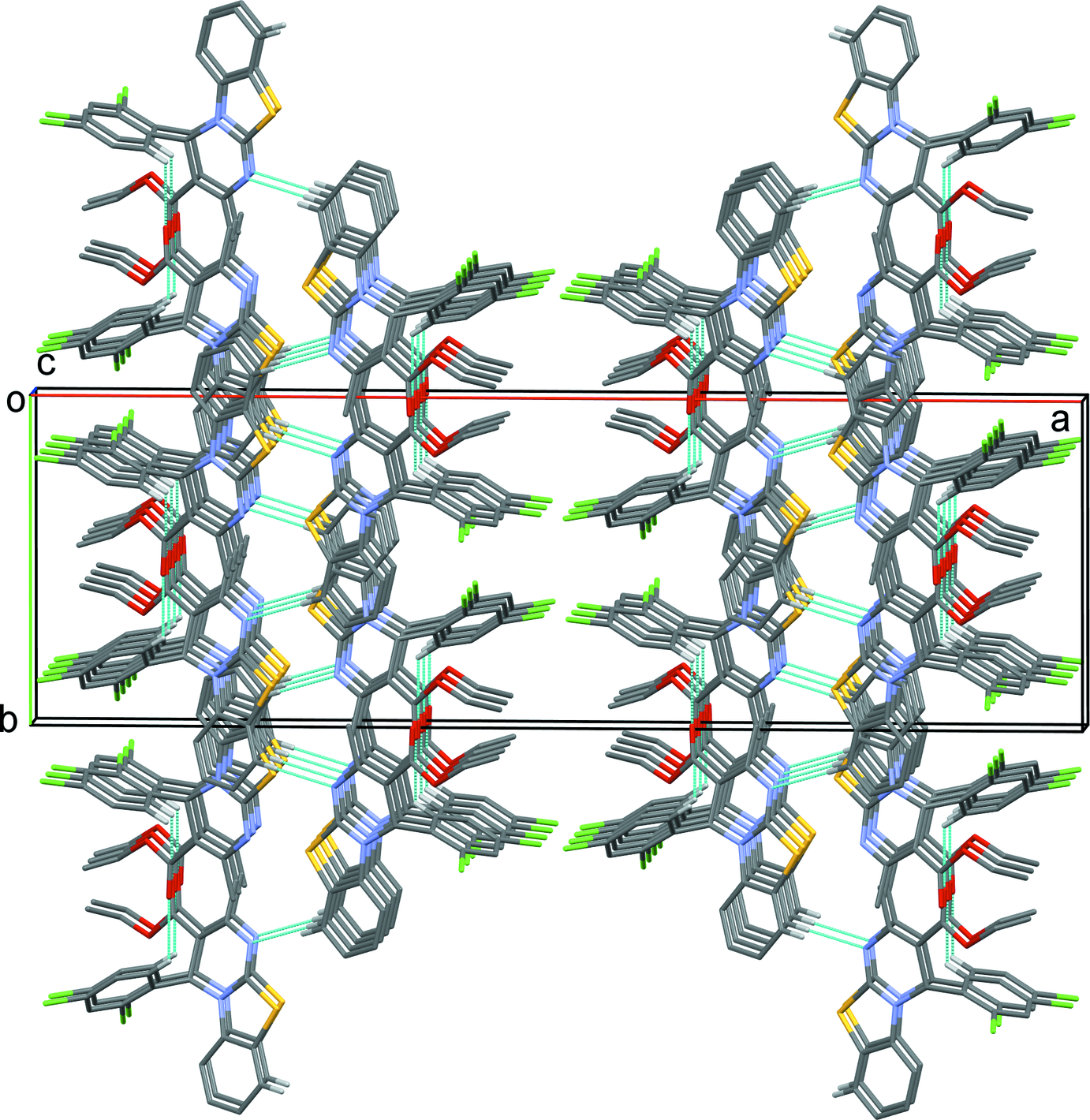

Supplement: Supplementary file 5 [file e-71-0o306-fig2.tif]
